# Supplementary material for: Paired Comparison of Routine Molecular Screening of Patient Samples with Advanced Non-Small Cell Lung Cancer in Circulating Cell-Free DNA Using Three Targeted Assays
Source: Cancers (Basel). 2023 Mar 3;15(5):1574. doi: 10.3390/cancers15051574 (PMC10001056; doi:10.3390/cancers15051574)
Supplement: Supplementary file 1 [file cancers-15-01574-s001.zip › cancers-2148910-supplementary.pdf]

Table S1. cfDNA molecular profiling of samples with discordant findings in at least one assay. * denotes actionable alterations; Bold denotes the *EGFR* gene. Status: (DI: at diagnosis; P: during progression; ND: Non Determined). The color gradient shows the Mutated Allelic Frequency (MAF): (Red: absence of alteration (MAF =0%); darker blue shows higher MAF levels).

ID Samples	Status (DI: at diagnosis; P: during progression)	Somatic alterations	MAF % Plasma-SeqSensei™ SOLID CANCER IVD kit	MAF % Plasma OncoBEAM™ EGFR V2	DNA content [ng input] Plasma-SeqSensei™ SOLID CANCER IVD kit	DNA content [ng input] on Plasma OncoBEAM™ EGFR V2	content [ng input] of Plasma OncoBEAMTM EGFR V2 /content [ng input] of Plasma-SeqSensei™ SOLID CANCER IVD kit
**2379**	**P**	**EGFRp.(Thr790Met)***	0.00	0.01	6.75	14.02	0.48
**1881**	**DI**	**EGFRp.(Leu858Arg)***	0.00	0.02	81.25	108.45	0.75
**2018**	**P**	**EGFRp.(Thr790Met)***	0.00	0.03	37.50	21.59	1.74
**1519**	**P**	**EGFRp.(Thr790Met)***	0.00	0.04	25.00	13.81	1.81
**2379**	**P**	**EGFRp.(Glu746_Ala750del)***	0.00	0.05	6.75	14.02	0.48
**1419**	**P**	**EGFRp.(Leu858Arg)***	0.00	0.05	25.00	18.28	1.37
**2045**	**P**	**EGFRp.(Thr790Met)***	0.00	0.05	5.89	9.35	0.63
**2045**	**P**	**EGFRp.(Glu746_Ala750del)***	0.00	0.05	5.89	9.35	0.63
**1827**	**P**	**EGFRp.(Glu746_Ala750del)***	0.00	0.05	6.29	6.23	1.01
**1622**	**P**	**EGFRp.(Leu858Arg)***	0.00	0.06	16.64	9.49	1.75
**1511**	**P**	**EGFRp.(Glu746_Ala750del)***	0.00	0.08	16.75	8.20	2.04
**1594**	**P**	**EGFRp.(Thr790Met)***	0.00	0.14	20.00	12.54	1.59
**1519**	**P**	**EGFRp.(Glu746_Ala750del)***	0.00	0.15	25.00	13.81	1.81
**1814**	**P**	**EGFRp.(Thr790Met)***	0.00	0.05	31.25	20.35	1.54
**1938**	**ND**	PIK3CA(p.His1047Arg)	0.06	0.00	85.00	5164.75	0.02
**2010**	**DI**	KRAS(p.Gly12Cys)*	0.08	0.00	36.81	58.70	0.63
**2105**	**P**	**EGFRp.(Leu718Gln)**	0.18	0.00	35.81	33.09	1.08
**2476**	**P**	**EGFRp.(Leu718Gln)**	0.18	0.00	36.81	31.88	1.15
**1966**	**DI**	PIK3CA(p.E545Q)	0.21	0.00	36.81	120.89	0.30
**2229**	**DI**	KRASp.(Gln61Arg)	0.25	0.00	30.25	31.60	0.96
**2724**	**P**	**EGFRp.(Ser768Ile)***	0.39	0.00	31.25	43.13	0.72

**1464**	**DI**	**EGFRp.(Ala871Gly)**	0.39	0.00	36.81	25.82	1.43
**2009**	**DI**	KRASp.(Gly12Asp)	0.50	0.00	85.00	72.72	1.17
**2460**	**DI**	BRAFp.(Val600Glu)*	0.50	0.00	15.62	30.51	0.51
**2428**	**P**	**EGFRp.(Leu718Gln)**	0.52	0.00	37.50	91.04	0.41
**2325**	**P**	KRASp.(Met72Leu)	0.55	0.00	15.45	12.37	1.25
**2453**	**DI**	BRAFp.(Val600Glu)*	0.59	0.00	6.24	10.39	0.60
**2547**	**P**	KRASp.(Met72Leu)	0.68	0.00	37.50	39.47	0.95
**2460**	**DI**	PIK3CAp.(Glu545Lys)	0.70	0.00	15.62	30.51	0.51
**2497**	**P**	KRASp.(Met72Leu)	0.97	0.00	23.66	23.05	1.03
**1962**	**DI**	PIK3CAp.(Glu545Gln)	1.14	0.00	36.81	110.80	0.33
**2463**	**DI**	KRASp.(Gly12Val)	1.20	0.00	26.87	25.65	1.05
**1603**	**ND**	**EGFRp.(Ser768Ile)***	1.21	0.00	14.14	11.05	1.28
**2455**	**DI**	**EGFRp.(Ile744.)**	1.40	0.00	16.87	18.92	0.89
**2019**	**DI**	BRAFp.(Val600Glu)*	1.72	0.00	14.63	22.36	0.65
**2455**	**DI**	PIK3CAp.(Glu542Lys)	1.90	0.00	16.87	18.92	0.89
**2268**	**ND**	**EGFRp.(Ser768Ile)***	2.37	0.00	25.98	46.31	0.56
**1887**	**P**	**EGFRp.(Ala767_Val769dup)***	4.13	0.00	31.25	25.35	1.23
**1564**	**DI**	KRASp.(Gly12Cys)*	10.23	0.00	36.81	8.51	4.32
**1845**	**DI**	KRASp.(Gly12Asp)	10.48	0.00	21.30	18.44	1.16
**1938**	**ND**	PIK3CAp.(Glu545Lys)	13.95	0.00	85.00	5164.75	0.02
**1895**	**DI**	PIK3CAp.(Glu545Lys)	15.14	0.00	43.75	48.71	0.90
**1870**	**DI**	KRASp.(Gly12Asp)	19.50	0.00	81.25	72.92	1.11
**1835**	**DI**	KRASp.(Gly12Asp)	21.17	0.00	75.00	56.59	1.33
**2336**	**DI**	PIK3CAp.(Glu545Lys)	27.58	0.00	36.88	37.69	0.98
**1579**	**DI**	KRASp.(Gly12Cys)*	35.81	0.00	36.81	157.20	0.23
**1901**	**DI**	KRASp.(Gly12Asp)	45.73	0.00	81.25	96.99	0.84

Table S2. CfDNA molecular profiling of samples with discordance findings in the custom-validated NGS assay and the Plasma-SeqSensei™ SOLID CANCER IVD assays of targeted genes of Plasma-SeqSensei™ SOLID CANCER IVD assay. * denotes actionable alterations; Bold denotes the *EGFR* gene. Status: (DI: at diagnosis; P: during progression; ND: Non Determined). The color gradient shows the Mutated Allelic Frequency (MAF): (Red: absence of alteration (MAF =0%); darker blue shows higher MAF levels).

ID Samples	Status (DI: at diagnosis; P: during progression)	Alteration	MAF % Plasma-SeqSensei™ SOLID CANCER IVD kit	MAF % custom-validated NGS assay	CfDNA input [ng input] of Plasma-SeqSensei™ SOLID CANCER IVD kit	CfDNA content [ng input] custom-validated NGS assay	Ratio of cfDNA content [ng input] of Plasma-SeqSensei™ SOLID CANCER IVD kit /cfDNA content [ng input]custom-validated NGS assay
2441	DI	KRASp.(Gly12Cys)*	0.00	0.50	3.44	8.49	0.41
2151	DI	PIK3CAp.(Arg524Lys)	0.00	1.40	11.56	35.00	0.33
2387	P	BRAFp.(Asp594Asn)	0.00	1.50	36.88	37.50	0.98
1843	DI	**EGFRp.(Leu62Arg)**	0.00	1.90	81.25	50.00	1.63
1962	DI	PIK3CAp.(Glu418Lys)	0.00	3.60	36.81	150.00	0.25
1845	DI	PIK3CAp.(Arg88Gln)	0.00	7.40	21.30	44.00	0.48
1517	ND	**EGFRp.(Val834Leu)**	0.00	14.40	36.81	50.00	0.74
1500	P	PIK3CAp.(Cys420Arg)	0.00	24.00	25.00	48.00	0.52
2338	DI	**EGFRp.(Gly779Cys)**	0.00	31.70	82.50	100.00	0.83
2025	P	**EGFRp.(Val843Ile)**	0.00	45.40	10.31	25.80	0.40
1596	p	**EGFRp.(Val834Leu)**	0.00	63.80	36.81	50.00	0.74
2909	P	**EGFRp.(Leu833Phe)**	0.00	85.60	37.50	100.00	0.38
2377	P	**EGFRp.(Glu746_Ala750del)***	0.02	0.00	36.81	72.30	0.51
1830	P	**EGFRp.(Leu858Arg)***	0.02	0.00	36.81	50.00	0.74
1938	ND	PIK3CA(p.His1047Arg)	0.06	0.00	85.00	150.00	0.57
2230	DI	**EGFRp.(Glu746_Ala750del)***	0.07	0.00	36.81	72.00	0.51
2010	DI	KRAS(p.Gly12Cys)*	0.08	0.00	36.81	117.50	0.31
2181	DI	**EGFRp.(Glu746_Ala750del)***	0.13	0.00	36.81	150.00	0.25

2105	P	**EGFRp.(Thr790Met)***	0.17	0.00	35.81	77.00	0.47
2105	P	**EGFRp.(Leu718Gln)**	0.18	0.00	35.81	77.00	0.47
2476	P	**EGFRp.(Leu718Gln)**	0.18	0.00	36.81	100.00	0.37
1740	P	**EGFRp.(Leu858Arg)***	0.19	0.00	25.00	50.00	0.50
2486	P	**EGFRp.(Cys797Ser)***	0.20	0.00	36.25	84.40	0.43
2909	P	**EGFRp.(Cys797Ser)***	0.20	0.00	37.50	100.00	0.38
1966	DI	PIK3CA(p.E545Q)	0.21	0.00	36.81	150.00	0.25
2229	DI	KRASp.(Gln61Arg)*	0.25	0.00	30.25	54.00	0.56
1594	P	**EGFRp.(Leu858Arg)***	0.28	0.00	20.00	30.00	0.67
2497	P	**EGFRp.(Leu858Arg)***	0.28	0.00	23.66	28.30	0.84
2912	DI	KRASp.(Gly12Asp)*	0.29	0.00	37.50	100.00	0.38
2547	P	**EGFRp.(Leu858Arg)***	0.30	0.00	37.50	37.00	1.01
2486	P	**EGFRp.(Thr790Met)***	0.30	0.00	36.25	84.40	0.43
2700	P	**EGFRp.(Leu861Gln)***	0.35	0.00	36.81	52.30	0.70
2876	DI	KRASp.(Lys117Asn)*	0.35	0.00	17.40	15.10	1.15
1601	P	**EGFRp.(Thr790Met)***	0.37	0.00	25.00	35.00	0.71
2724	P	**EGFRp.(Ser768Ile)***	0.39	0.00	31.25	66.00	0.47
2476	P	**EGFRp.(Thr790Met)***	0.43	0.00	36.81	100.00	0.37
2428	P	**EGFRp.(Leu718Gln)**	0.52	0.00	37.50	100.00	0.38
2476	P	**EGFRp.(Leu858Arg)***	0.61	0.00	36.81	100.00	0.37
2547	P	KRASp.(Met72Leu)*	0.68	0.00	37.50	37.00	1.01
2428	P	**EGFRp.(Thr790Met)***	0.83	0.00	37.50	100.00	0.38
2017	P	**EGFRp.(Thr790Met)***	0.91	0.00	85.00	146.00	0.58
1800	P	**EGFRp.(Glu746_Ala750del)***	0.92	0.00	7.38	16.50	0.45
2464	P	**EGFRp.(Thr790Met)***	1.20	0.00	85.00	150.00	0.57
2455	DI	**EGFRp.(Ile744.)**	1.40	0.00	16.87	34.80	0.48

Table S3. CfDNA molecular profiling of samples with discordance findings in custom-validated NGS and Plasma-SeqSensei™ NSCLC IVDR assays of other targeted genes. Status: (DI: at diagnosis; P: during progression; ND: Non Determined). The color gradient shows the Mutated Allelic Frequency (MAF): (Red: absence of alteration (MAF =0%); darker blue shows higher MAF levels).

ID Samples	Status (DI: at diagnosis; P: during progression; ND: unknown)	Alteration	MAF % custom-validated NGS assay	MAF % Plasma-SeqSensei™ SOLID CANCER IVD kit	CfDNA input [ng input] of Plasma-SeqSensei™ SOLID CANCER IVD kit	CfDNA content [ng input] custom-validated NGS assay	Ratio of cfDNA content [ng input] of Plasma-SeqSensei™ SOLID CANCER IVD kit /cfDNA content [ng input]custom-validated NGS assay
2934	P	**TP53p.(Val172Phe)**	0.40	0.00	20.00	20.50	0.98
2018	P	**TP53p.(Arg175His)**	0.80	0.00	37.50	61.00	0.61
1529	DI	ARID1Ap.(Thr1888Valfs*36)	1.30	0.00	36.81	50.00	0.74
2229	DI	**TP53p.(Cys135Tyr)**	1.40	0.00	30.25	54.00	0.56
2455	DI	**METp.(?)**	1.50	0.00	16.87	34.80	0.48
1984	P	**TP53p.(Arg248Trp)**	1.50	0.00	81.25	150.00	0.54
2485	P	**TP53p.(Arg248Trp)**	1.60	0.00	10.05	50.00	0.20
2932	DI	**TP53p.(Ile255del)**	1.60	0.00	36.25	75.00	0.48
2919	P	**TP53p.(Ile255Phe)**	1.60	0.00	36.81	59.70	0.62
1608	P	**TP53p.(Gln136*)**	1.64	0.00	36.81	50.00	0.74
2909	P	**TP53p.(Val157Phe)**	1.80	0.00	37.50	100.00	0.38
1647	P	NRAS c.291-131_450+328del	1.90	0.00	16.75	31.00	0.54
1862	DI	**TP53p.(Trp91Glyfs*32)**	2.44	0.00	36.81	16.00	2.30
2935	DI	**TP53p.(Arg248Trp)**	2.50	0.00	25.00	30.40	0.82
2019	DI	NRASp.(?)	2.70	0.00	14.63	25.50	0.57
2275	P	**TP53p.(Val274Ala)**	2.80	0.00	18.29	13.75	1.33
2941	DI	**TP53p.(Ser215Gly)**	3.00	0.00	16.75	28.30	0.59
2893	P	NOTCH1p.(Pro2415del)	3.10	0.00	33.99	26.80	1.27
2030	DI	**TP53p.(Cys176Phe)**	3.20	0.00	59.25	107.80	0.55
2155	DI	**TP53p.(Arg213*)**	3.80	0.00	36.88	107.50	0.34
2103	DI	CTNNB1p.(Ala39_Ala43del)	4.30	0.00	36.88	85.00	0.43
1517	ND	**TP53p.(Lys132Arg)**	4.50	0.00	36.81	50.00	0.74
2931	P	**TP53p.(Glu224*)**	5.40	0.00	36.25	57.70	0.63
2455	DI	**METp.(Leu1213Phe)**	5.90	0.00	16.87	34.80	0.48

2053	P	**TP53p.(Arg110Leu)**	6.10	0.00	17.50	44.20	0.40
1606	P	**TP53p.(Gln375*)**	6.54	0.00	36.81	50.00	0.74
2919	P	GNASp.(Arg201His)	7.90	0.00	36.81	59.70	0.62
2472	P	**TP53p.(?)**	8.10	0.00	85.00	93.00	0.91
2921	P	**TP53p.(Gln331*)**	8.10	0.00	36.25	73.70	0.49
2941	DI	**TP53p.(Cys275Tyr)**	9.10	0.00	16.75	28.30	0.59
2155	DI	**TP53p.(Arg333Valfs*12)**	9.20	0.00	36.88	107.50	0.34
2061	P	CDKN2Ap.(Ser56Argfs*56)	9.70	0.00	79.38	150.00	0.53
1901	DI	**TP53p.(Val147Glyfs*2)**	9.90	0.00	81.25	150.00	0.54
1599	DI	SMAD4p.(Trp302*)	10.00	0.00	36.81	50.00	0.74
2455	DI	**TP53p.(Glu294Glyfs*12)**	11.30	0.00	16.87	34.80	0.48
1845	DI	**TP53p.(Val272Leu)**	12.70	0.00	21.30	44.00	0.48
2937	DI	H3F3Ap.(Ser29Thr)	13.20	0.00	12.50	20.50	0.61
1614	P	ERBB4p.(Val391Ala)	17.90	0.00	36.81	50.00	0.74
2338	DI	**TP53p.(Val173Leu)**	24.60	0.00	82.50	100.00	0.83
2915	DI	**TP53p.(His179Tyr)**	26.20	0.00	37.50	36.20	1.04
1619	P	**ERBB2p.(Ile654_Ile655delinsValVal)**	35.60	0.00	36.81	50.00	0.74
1556	ND	ERBB4p.(Val391Ala)	37.10	0.00	36.81	50.00	0.74
2905	P	**TP53p.(Arg158Pro)**	37.20	0.00	36.81	100.00	0.37
2929	DI	**TP53p.(Asn131del)**	38.70	0.00	36.25	100.00	0.36
1620	ND	**ERBB2p.(Ile654_Ile655delinsValVal)**	38.90	0.00	36.81	50.00	0.74
1606	P	**METp.(Arg988Cys)**	46.77	0.00	36.81	50.00	0.74
1938	ND	**TP53p.(Arg273Cys)**	47.20	0.00	85.00	150.00	0.57
2061	P	**TP53p.(Arg283Cys)**	56.50	0.00	79.38	150.00	0.53
1596	p	**TP53p.(Lys132Arg)**	62.10	0.00	36.81	50.00	0.74

Table S4. Complete CfDNA molecular profiling of samples with the three assays. Status: (DI: at diagnosis; P: during progression; ND: Non Determined).

ID Samples	Status	Date SafeSeq	Gene	Alteration	Plasma-SeqSensei™ SOLID CANCER IVD kit	MAF % Plasma-SeqSensei™ SOLID CANCER IVD kit	Plasma SeqSensei cfDNA input	Alteration Plasma OncoBEAMTM EGFR V2	MAF % Plasma OncoBEAM™ EGFR V2	BEAMing DNA content [ng input]	Alteration custom-validated NGS assay	MAF % custom-validated NGS assay	Sample DNA content [ng]NGS-SOPHIA
721	P	28/07/2022	**Wild-type (WT)**	**Wild-type (WT)**	WT	0.00	19.68	NR	NR	NR	WT	0.00	16.00
1403	P	20/07/2022	**Wild-type (WT)**	**Wild-type (WT)**	WT	0.00	30.00	NR	NR	NR	WT	0.00	25.00
1419	P	04/07/2022	**EGFR**	**EGFRp.(Leu858Arg)**	WT	0.00	25.00	EGFRp.(Leu858Arg)	0.05	18.28	WT	0.00	50.00
1430	ND	20/07/2022	**Wild-type (WT)**	**Wild-type (WT)**	WT	0.00	26.50	NR	NR	NR	WT	0.00	20.00
1464	DI	20/07/2022	**EGFR**	**EGFRp.(Leu858Arg)**	EGFRp.(Leu858Arg)	0.38	36.81	EGFRp.(Leu858Arg)	0.11	25.82	EGFRp.(Leu858Arg)	0.43	12.50
1464	DI	20/07/2022	**EGFR**	**EGFRp.(Ala871Gly)**	EGFRp.(Ala871Gly)	0.39	36.81	WT	0.00	25.82	EGFRp.(Ala871Gly)	0.42	12.50
1480	P	04/07/2022	**Wild-type (WT)**	**Wild-type (WT)**	WT	0.00	36.81	NR	NR	NR	WT	0.00	50.00
1500	P	04/07/2022	**PIK3CA**	**PIK3CAp.(Cys420Arg)**	WT	0.00	25.00	WT	0.00	12.78	PIK3CAp.(Cys420Arg)	24.00	48.00
1500	P	04/07/2022	**EGFR**	**EGFRp.(Thr790Met)**	EGFRp.(Thr790Met)	8.48	25.00	EGFRp.(Thr790Met)	3.98	12.78	EGFRp.(Thr790Met)	7.90	48.00
1500	P	04/07/2022	**EGFR**	**EGFRp.(Glu746_Ala750del)**	EG-FRp.(Glu746_Ala750del)	12.78	25.00	EGFRp.(Glu746_Ala750del)	11.63	12.78	EG-FRp.(Glu746_Ala750del)	14.80	48.00
1503	P	28/07/2022	**Wild-type (WT)**	**Wild-type (WT)**	WT	0.00	13.65	WT	0.00	14.90	WT	0.00	35.00
1511	P	04/07/2022	**EGFR**	**EGFRp.(Glu746_Ala750del)**	WT	0.00	16.75	EGFRp.(Glu746_Ala750del)	0.08	8.20	WT	0.00	27.00
1512	P	28/07/2022	**Wild-type (WT)**	**Wild-type (WT)**	WT	0.00	7.41	WT	0.00	7.04	WT	0.00	20.00
1517	ND	04/07/2022	**Other alterations**	**TP53p.(Lys132Arg)**	WT	0.00	36.81	WT	0.00	32.66	TP53p.(Lys132Arg)	4.50	50.00
1517	ND	04/07/2022	**EGFR**	**EGFRp.(Val834Leu)**	WT	0.00	36.81	WT	0.00	32.66	EGFRp.(Val834Leu)	14.40	50.00
1517	ND	04/07/2022	**EGFR**	**EGFRp.(Leu858Arg)**	EGFRp.(Leu858Arg)	13.22	36.81	EGFRp.(Leu858Arg)	5.99	32.66	EGFRp.(Leu858Arg)	15.60	50.00
1518	P	04/07/2022	**EGFR**	**EGFRp.(Leu858Arg)**	EGFRp.(Leu858Arg)	0.26	36.81	EGFRp.(Leu858Arg)	0.13	41.55	EGFRp.(Leu858Arg)	0.70	50.00
1519	P	04/07/2022	**EGFR**	**EGFRp.(Thr790Met)**	WT	0.00	25.00	EGFRp.(Thr790Met)	0.04	13.81	WT	0.00	35.00
1519	P	04/07/2022	**EGFR**	**EGFRp.(Glu746_Ala750del)**	WT	0.00	25.00	EGFRp.(Glu746_Ala750del)	0.15	13.81	WT	0.00	35.00
1525	P	03/08/2022	**Wild-type (WT)**	**Wild-type (WT)**	WT	0.00	2.10	WT	0.00	6.65	WT	0.00	12.00
1528	DI	04/07/2022	**EGFR**	**EGFRp.(Leu861Gln)**	EGFRp.(Leu861Gln)	5.58	16.75	EGFRp.(Leu861Gln)	3.23	8.31	EGFRp.(Leu861Gln)	8.10	30.00
1529	DI	04/07/2022	**Other alterations**	**ARID1Ap.(Thr1888Valfs*36)**	WT	0.00	36.81	WT	0.00	11.47	ARID1Ap.(Thr1888Valfs*36)	1.30	50.00
1529	DI	04/07/2022	**EGFR**	**EGFRp.(Leu858Arg)**	EGFRp.(Leu858Arg)	1.50	36.81	EGFRp.(Leu858Arg)	0.88	11.47	EGFRp.(Leu858Arg)	2.40	50.00
1533	P	04/07/2022	**EGFR**	**EGFRp.(Glu746_Ala750del)**	EG-FRp.(Glu746_Ala750del)	1.69	20.00	EGFRp.(Glu746_Ala750del)	1.40	7.15	EG-FRp.(Glu746_Ala750del)	1.60	35.00
1534	DI	20/07/2022	**Wild-type (WT)**	**Wild-type (WT)**	WT	0.00	9.35	WT	0.00	4.19	WT	0.00	15.00
1546	p	04/07/2022	**EGFR**	**EGFRp.(Glu746_Ala750del)**	EG-FRp.(Glu746_Ala750del)	1.45	10.63	EGFRp.(Glu746_Ala750del)	1.63	10.29	EG-FRp.(Glu746_Ala750del)	1.20	15.00
1547	P	04/07/2022	**EGFR**	**EGFRp.(Gly719Ala)**	EGFRp.(Gly719Ala)	0.48	20.00	EGFRp.(Gly719X)	0.45	18.00	EGFRp.(Gly719Ala)	1.00	30.00
1556	ND	04/07/2022	**Other alterations**	**ERBB4p.(Val391Ala)**	WT	0.00	36.81	WT	0.00	21.76	ERBB4p.(Val391Ala)	37.10	50.00
1562	ND	04/07/2022	**EGFR**	**EGFRp.(Leu858Arg)**	EGFRp.(Leu858Arg)	0.35	25.00	EGFRp.(Leu858Arg)	0.36	5.35	EGFRp.(Leu858Arg)	1.10	50.00
1564	DI	04/07/2022	**KRAS**	**KRASp.(Gly12Cys)**	KRASp.(Gly12Cys)	10.23	36.81	WT	0.00	8.51	KRASp.(Gly12Cys)	13.00	50.00
1571	DI	13/07/2022	**EGFR**	**EGFRp.(Leu858Arg)**	EGFRp.(Leu858Arg)	0.80	23.36	EGFRp.(Leu858Arg)	0.41	12.41	EGFRp.(Leu858Arg)	0.90	35.00
1572	DI	13/07/2022	**Wild-type (WT)**	**Wild-type (WT)**	WT	0.00	36.81	WT	0.00	25.79	WT	0.00	50.00
1574	P	01/08/2022	**Wild-type (WT)**	**Wild-type (WT)**	WT	0.00	3.93	WT	0.00	6.44	WT	0.00	15.00
1577	P	13/07/2022	**EGFR**	**EGFRp.(Glu746_Ala750del)**	EG-FRp.(Glu746_Ala750del)	0.27	16.01	EGFRp.(Glu746_Ala750del)	0.26	7.21	EG-FRp.(Glu746_Ala750del)	16.60	26.00
1578	DI	13/07/2022	**EGFR**	**EGFRp.(Leu858Arg)**	EGFRp.(Leu858Arg)	0.34	36.81	EGFRp.(Leu858Arg)	0.19	26.92	EGFRp.(Leu858Arg)	0.40	50.00
1579	DI	13/07/2022	**KRAS**	**KRASp.(Gly12Cys)**	KRASp.(Gly12Cys)	35.81	36.81	WT	0.00	157.20	KRASp.(Gly12Cys)	38.00	50.00
1592	P	28/07/2022	**Wild-type (WT)**	**Wild-type (WT)**	WT	0.00	7.65	WT	0.00	7.04	WT	0.00	17.00
1594	P	13/07/2022	**EGFR**	**EGFRp.(Thr790Met)**	WT	0.00	20.00	EGFRp.(Thr790Met)	0.14	12.54	WT	0.00	30.00
1594	P	13/07/2022	**EGFR**	**EGFRp.(Leu858Arg)**	EGFRp.(Leu858Arg)	0.28	20.00	EGFRp.(Leu858Arg)	0.09	12.54	WT	0.00	30.00
1596	p	13/07/2022	**Other alterations**	**TP53p.(Lys132Arg)**	WT	0.00	36.81	NR	0.00	155.03	TP53p.(Lys132Arg)	62.10	50.00
1596	p	13/07/2022	**EGFR**	**EGFRp.(Val834Leu)**	WT	0.00	36.81	WT	0.00	155.03	EGFRp.(Val834Leu)	63.80	50.00
1596	p	13/07/2022	**EGFR**	**EGFRp.(Thr790Met)**	EGFRp.(Thr790Met)	1.94	36.81	EGFRp.(Thr790Met)	1.02	155.03	EGFRp.(Thr790Met)	1.50	50.00
1596	p	13/07/2022	**EGFR**	**EGFRp.(Leu858Arg)**	EGFRp.(Leu858Arg)	56.19	36.81	EGFRp.(Leu858Arg)	40.11	155.03	EGFRp.(Leu858Arg)	61.60	50.00
1597	DI	20/07/2022	**Wild-type (WT)**	**Wild-type (WT)**	WT	0.00	18.75	WT	0.00	14.84	WT	0.00	36.00
1599	DI	20/07/2022	**Other alterations**	**SMAD4p.(Trp302*)**	WT	0.00	36.81	WT	0.00	71.17	SMAD4p.(Trp302*)	10.00	50.00
1600	ND	13/07/2022	**EGFR**	**EGFRp.(Thr790Met)**	EGFRp.(Thr790Met)	0.70	25.00	EGFRp.(Thr790Met)	0.58	14.52	EGFRp.(Thr790Met)	0.50	35.00
1600	ND	13/07/2022	**EGFR**	**EGFRp.(Leu861Gln)**	EGFRp.(Leu861Gln)	1.69	25.00	EGFRp.(Leu861Gln)	1.73	14.52	EGFRp.(Leu861Gln)	1.80	35.00
1601	P	13/07/2022	**EGFR**	**EGFRp.(Thr790Met)**	EGFRp.(Thr790Met)	0.37	25.00	EGFRp.(Thr790Met)	0.06	16.50	WT	0.00	35.00

1603	ND	13/07/2022	**EGFR**	**EGFRp.(Leu858Arg)**	EGFRp.(Leu858Arg)	1.13	14.14	EGFRp.(Leu858Arg)	0.74	11.05	EGFRp.(Leu858Arg)	1.46	23.00
1603	ND	13/07/2022	**EGFR**	**EGFRp.(Ser768Ile)**	EGFRp.(Ser768Ile)	1.21	14.14	WT	0.00	11.05	EGFRp.(Ser768Ile)	1.41	23.00
1606	P	20/07/2022	**Other alterations**	**TP53p.(Gln375*)**	WT	0.00	36.81	NR	NR	NR	TP53p.(Gln375*)	6.54	50.00
1606	P	20/07/2022	**Other alterations**	**METp.(Arg988Cys)**	WT	0.00	36.81	NR	NR	NR	METp.(Arg988Cys)	46.77	50.00
1606	P	20/07/2022	**EGFR**	**EGFRp.(Leu747Ser)**	EGFRp.(Leu747Ser)	3.37	36.81	NR	NR	NR	EGFRp.(Leu747Ser)	8.41	50.00
1608	P	13/07/2022	**Other alterations**	**TP53p.(Gln136*)**	WT	0.00	36.81	WT	0.00	58.29	TP53p.(Gln136*)	1.64	50.00
1608	P	13/07/2022	**EGFR**	**EGFRp.(Leu858Arg)**	EGFRp.(Leu858Arg)	1.20	36.81	EGFRp.(Leu858Arg)	0.78	58.29	EGFRp.(Leu858Arg)	1.06	50.00
1614	P	13/07/2022	**Other alterations**	**ERBB4p.(Val391Ala)**	WT	0.00	36.81	WT	0.00	21.80	ERBB4p.(Val391Ala)	17.90	50.00
1619	P	28/07/2022	**Other alterations**	**ERBB2p.(Ile654_Ile655de-linsValVal)**	WT	0.00	36.81	WT	0.00	61.15	ERBB2p.(Ile654_Ile655de-linsValVal)	35.60	50.00
1620	ND	13/07/2022	**Other alterations**	**ERBB2p.(Ile654_Ile655de-linsValVal)**	WT	0.00	36.81	WT	0.00	68.33	ERBB2p.(Ile654_Ile655de-linsValVal)	38.90	50.00
1620	ND	13/07/2022	**EGFR**	**EGFRp.(Thr790Met)**	EGFRp.(Thr790Met)	6.59	36.81	EGFRp.(Thr790Met)	3.45	68.33	EGFRp.(Thr790Met)	5.10	50.00
1620	ND	13/07/2022	**EGFR**	**EGFRp.(Leu858Arg)**	EGFRp.(Leu858Arg)	14.14	36.81	EGFRp.(Leu858Arg)	9.53	68.33	EGFRp.(Leu858Arg)	11.90	50.00
1622	P	13/07/2022	**EGFR**	**EGFRp.(Leu858Arg)**	WT	0.00	16.64	EGFRp.(Leu858Arg)	0.06	9.49	WT	0.00	50.00
1635	P	13/07/2022	**EGFR**	**EGFRp.(Leu861Gln)**	EGFRp.(Leu861Gln)	1.69	11.77	EGFRp.(Leu861Gln)	0.27	11.69	EGFRp.(Leu861Gln)	1.20	23.70
1647	P	18/07/2022	**Other alterations**	**NRAS c.291-131_450+328del**	WT	0.00	16.75	WT	0.00	11.71	NRAS c.291-131_450+328del	1.90	31.00
1647	P	18/07/2022	**EGFR**	**EGFRp.(Glu746_Ala750del)**	EG-FRp.(Glu746_Ala750del)	0.45	16.75	EGFRp.(Glu746_Ala750del)	0.30	11.71	NR	NR	31.00
1660	P	01/08/2022	**Wild-type (WT)**	**Wild-type (WT)**	WT	0.00	2.55	WT	0.00	5.89	WT	0.00	11.75
1709	P	01/08/2022	**Wild-type (WT)**	**Wild-type (WT)**	WT	0.00	3.69	WT	0.00	4.49	WT	0.00	15.00
1740	P	18/07/2022	**EGFR**	**EGFRp.(Leu858Arg)**	EGFRp.(Leu858Arg)	0.19	25.00	EGFRp.(Leu858Arg)	0.07	17.80	WT	0.00	50.00
1774	DI	14/05/2021	**KRAS**	**KRASp.(Gly12Cys)**	KRASp.(Gly12Cys)	5.30	50.00	NR	NR	NR	KRASp.(Gly12Cys)	4.90	50.00
1777	DI	14/05/2021	**EGFR**	**EGFRp.(Glu746_Ala750del)**	EG-FRp.(Glu746_Ala750del)	1.93	36.87	NR	NR	NR	EG-FRp.(Glu746_Ala750del)	2.30	43.00
1800	P	18/07/2022	**EGFR**	**EGFRp.(Glu746_Ala750del)**	EG-FRp.(Glu746_Ala750del)	0.92	7.38	EGFRp.(Glu746_Ala750del)	0.58	10.24	WT	0.00	16.50
1814	P	03/06/2021	**EGFR**	**EGFRp.(Thr790Met)**	WT	0.00	31.25	EGFRp.(Thr790Met)	0.05	20.35	NR	NR	NR
1814	P	03/06/2021	**EGFR**	**EGFRp.(Leu861Gln)**	EGFRp.(Leu861Gln)	0.22	31.25	EGFRp.(Leu861Gln)	0.06	20.35	NR	NR	NR
1827	P	17/08/2022	**EGFR**	**EGFRp.(Glu746_Ala750del)**	WT	0.00	6.29	EGFRp.(Glu746_Ala750del)	0.05	6.23	WT	0.00	15.10
1830	P	18/07/2022	**EGFR**	**EGFRp.(Leu858Arg)**	EGFRp.(Leu858Arg)	0.02	36.81	EGFRp.(Leu858Arg)	0.04	32.85	WT	0.00	50.00
1835	DI	14/05/2021	**KRAS**	**KRASp.(Gly12Asp)**	KRASp.(Gly12Asp)	21.17	75.00	WT	0.00	56.59	KRASp.(Gly12Asp)	21.40	50.00
1843	DI	14/05/2021	**EGFR**	**EGFRp.(Leu62Arg)**	WT	0.00	81.25	WT	0.00	42.14	EGFRp.(Leu62Arg)	1.90	50.00
1843	DI	14/05/2021	**EGFR**	**EGFRp.(Leu858Arg)**	EGFRp.(Leu858Arg)	2.78	81.25	EGFRp.(Leu858Arg)	1.34	42.14	EGFRp.(Leu858Arg)	2.30	50.00
1845	DI	02/08/2021	**PIK3CA**	**PIK3CAp.(Arg88Gln)**	WT	0.00	21.30	WT	0.00	18.44	PIK3CAp.(Arg88Gln)	7.40	44.00
1845	DI	02/08/2021	**Other alterations**	**TP53p.(Val272Leu)**	WT	0.00	21.30	NR	0.00	18.44	TP53p.(Val272Leu)	12.70	44.00
1845	DI	02/08/2021	**KRAS**	**KRASp.(Gly12Asp)**	KRASp.(Gly12Asp)	10.48	21.30	WT	0.00	18.44	KRASp.(Gly12Asp)	14.00	44.00
1846	DI	14/05/2021	**EGFR**	**EGFRp.(Glu746_Ala750del)**	EG-FRp.(Glu746_Ala750del)	2.73	81.25	EGFRp.(Glu746_Ala750del)	2.69	25.20	EG-FRp.(Glu746_Ala750del)	2.80	50.00
1862	DI	20/07/2022	**Other alterations**	**TP53p.(Trp91Glyfs*32)**	WT	0.00	36.81	WT	0.00	17.34	TP53p.(Trp91Glyfs*32)	2.44	16.00
1867	DI	14/05/2021	**EGFR**	**EGFRp.(Leu858Arg)**	EGFRp.(Leu858Arg)	0.18	56.75	EGFRp.(Leu858Arg)	0.27	36.07	EGFRp.(Leu858Arg)	0.70	50.00
1870	DI	14/05/2021	**KRAS**	**KRASp.(Gly12Asp)**	KRASp.(Gly12Asp)	19.50	81.25	WT	0.00	72.92	KRASp.(Gly12Asp)	17.00	50.00
1876	DI	20/07/2022	**Wild-type (WT)**	**Wild-type (WT)**	WT	0.00	36.81	WT	0.00	24.89	WT	0.00	66.00
1881	DI	14/05/2021	**EGFR**	**EGFRp.(Leu858Arg)**	WT	0.00	81.25	EGFRp.(Leu858Arg)	0.02	108.45	WT	0.00	50.00
1887	P	14/05/2021	**EGFR**	**EGFRp.(Ala767_Val769dup)**	EG-FRp.(Ala767_Val769dup)	4.13	31.25	WT	0.00	25.35	EG-FRp.(Ala767_Val769dup)	5.60	75.00
1889	P	02/08/2021	**EGFR**	**EGFRp.(Cys797Ser)**	EGFRp.(Cys797Ser)	4.12	25.00	EGFRp.(Cys797Ser)	4.83	22.43	EGFRp.(Cys797Ser)	6.30	40.80
1889	P	02/08/2021	**EGFR**	**EGFRp.(Thr790Met)**	EGFRp.(Thr790Met)	20.30	25.00	EGFRp.(Thr790Met)	9.36	22.43	EGFRp.(Thr790Met)	20.60	40.80
1889	P	02/08/2021	**EGFR**	**EGFRp.(Leu747_Ala750de-linsPro)**	EG-FRp.(Leu747_Ala750de-linsPro)	45.70	25.00	EGFRp.(Glu746_Ala750del)	23.65	22.43	EG-FRp.(Leu747_Ala750de-linsPro)	45.70	40.80
1892	P	02/08/2021	**EGFR**	**EGFRp.(Leu861Gln)**	EGFRp.(Leu861Gln)	2.04	10.63	EGFRp.(Leu861Gln)	0.82	9.88	EGFRp.(Leu861Gln)	1.60	16.00
1895	DI	14/05/2021	**PIK3CA**	**PIK3CAp.(Glu545Lys)**	PIK3CAp.(Glu545Lys)	15.14	43.75	WT	0.00	48.71	PIK3CAp.(Glu545Lys)	1.40	102.30
1901	DI	14/05/2021	**Other alterations**	**TP53p.(Val147Glyfs*2)**	WT	0.00	81.25	WT	0.00	96.99	TP53p.(Val147Glyfs*2)	9.90	150.00
1901	DI	14/05/2021	**KRAS**	**KRASp.(Gly12Asp)**	KRASp.(Gly12Asp)	45.73	81.25	WT	0.00	96.99	KRASp.(Gly12Asp)	44.70	150.00
1905	DI	20/07/2022	**Wild-type (WT)**	**Wild-type (WT)**	WT	0.00	36.81	WT	0.00	76.42	WT	0.00	150.00
1913	P	01/08/2022	**Wild-type (WT)**	**Wild-type (WT)**	WT	0.00	4.95	WT	0.00	7.40	WT	0.00	11.15
1938	ND	02/08/2021	**Other alterations**	**TP53p.(Arg273Cys)**	WT	0.00	85.00	WT	0.00	5164.75	TP53p.(Arg273Cys)	47.20	150.00
1938	ND	02/08/2021	**PIK3CA**	**PIK3CA(p.His1047Arg)**	PIK3CA(p.His1047Arg)	0.06	85.00	WT	0.00	5164.75	WT	0.00	150.00

1938	ND	02/08/2021	**PIK3CA**	**PIK3CAp.(Glu545Lys)**	PIK3CAp.(Glu545Lys)	13.95	85.00	WT	0.00	5164.75	PIK3CAp.(Glu545Lys)	13.80	150.00
1938	ND	02/08/2021	**EGFR**	**EGFRp.(Glu746_Thr751de-linsVal)**	EG-FRp.(Glu746_Thr751de-linsVal)	40.35	85.00	EGFRp.(Glu746_Ala750del)	37.57	5164.75	EG-FRp.(Glu746_Thr751de-linsVal)	38.90	150.00
1962	DI	20/07/2022	**PIK3CA**	**PIK3CAp.(Glu418Lys)**	WT	0.00	36.81	WT	0.00	110.80	PIK3CAp.(Glu418Lys)	3.60	150.00
1962	DI	20/07/2022	**PIK3CA**	**PIK3CAp.(Glu545Gln)**	PIK3CAp.(Glu545Gln)	1.14	36.81	WT	0.00	110.80	PIK3CAp.(Glu545Gln)	2.20	150.00
1962	DI	20/07/2022	**EGFR**	**EGFRp.(Leu858Arg)**	EGFRp.(Leu858Arg)	29.44	36.81	EGFRp.(Leu858Arg)	19.93	110.80	EGFRp.(Leu858Arg)	34.30	150.00
1966	DI	20/07/2022	**PIK3CA**	**PIK3CA(p.E545Q)**	PIK3CA(p.E545Q)	0.21	36.81	WT	0.00	120.89	WT	0.00	150.00
1966	DI	20/07/2022	**EGFR**	**EGFRp.(Leu858Arg)**	EGFRp.(Leu858Arg)	14.70	36.81	EGFRp.(Leu858Arg)	10.18	120.89	EGFRp.(Leu858Arg)	15.80	150.00
1979	DI	20/07/2022	**Wild-type (WT)**	**Wild-type (WT)**	WT	0.00	36.81	WT	0.00	259.47	WT	0.00	150.00
1984	P	17/08/2022	**Other alterations**	**TP53p.(Arg248Trp)**	WT	0.00	81.25	WT	0.00	65.16	TP53p.(Arg248Trp)	1.50	150.00
1984	P	17/08/2022	**EGFR**	**EGFRp.(Thr790Met)**	EGFRp.(Thr790Met)	0.51	81.25	EGFRp.(Thr790Met)	0.34	65.16	EGFRp.(Thr790Met)	0.52	150.00
1984	P	17/08/2022	**EGFR**	**EGFRp.(Leu858Arg)**	EGFRp.(Leu858Arg)	29.06	81.25	EGFRp.(Leu858Arg)	19.38	65.16	EGFRp.(Leu858Arg)	32.60	150.00
1993	ND	20/07/2022	**BRAF**	**BRAFp.(Val600Glu)**	BRAFp.(Val600Glu)	2.49	21.56	NR	NR	NR	BRAFp.(Val600Glu)	4.00	15.25
1997	DI	28/07/2022	**Wild-type (WT)**	**Wild-type (WT)**	WT	0.00	9.09	WT	0.00	12.34	WT	0.00	24.25
2009	DI	02/08/2021	**KRAS**	**KRASp.(Gly12Asp)**	KRASp.(Gly12Asp)	0.50	85.00	WT	0.00	72.72	KRASp.(Gly12Asp)	0.50	150.00
2010	DI	28/07/2022	**KRAS**	**KRAS(p.Gly12Cys)**	KRAS(p.Gly12Cys)	0.08	36.81	WT	0.00	58.70	WT	0.00	117.50
2012	ND	20/07/2022	**Wild-type (WT)**	**Wild-type (WT)**	WT	0.00	20.31	NR	NR	NR	WT	0.00	14.50
2017	P	02/08/2021	**EGFR**	**EGFRp.(Thr790Met)**	EGFRp.(Thr790Met)	0.91	85.00	EGFRp.(Thr790Met)	0.51	84.47	WT	0.00	146.00
2017	P	02/08/2021	**EGFR**	**EGFRp.(Leu858Arg)**	EGFRp.(Leu858Arg)	1.33	85.00	EGFRp.(Leu858Arg)	0.86	84.47	EGFRp.(Leu858Arg)	1.60	146.00
2018	P	03/06/2021	**EGFR**	**EGFRp.(Thr790Met)**	WT	0.00	37.50	EGFRp.(Thr790Met)	0.03	21.59	WT	0.00	61.00
2018	P	03/06/2021	**Other alterations**	**TP53p.(Arg175His)**	WT	0.00	37.50	WT	0.00	21.59	TP53p.(Arg175His)	0.80	61.00
2018	P	03/06/2021	**EGFR**	**EGFRp.(Leu861Gln)**	EGFRp.(Leu861Gln)	1.53	37.50	EGFRp.(Leu861Gln)	1.29	21.59	EGFRp.(Leu861Gln)	2.20	61.00
2019	DI	02/08/2021	**Other alterations**	**NRASp.(?)**	WT	0.00	14.63	WT	0.00	22.36	NRASp.(?)	2.70	25.50
2019	DI	02/08/2021	**BRAF**	**BRAFp.(Val600Glu)**	BRAFp.(Val600Glu)	1.72	14.63	WT	0.00	22.36	BRAFp.(Val600Glu)	1.40	25.50
2025	P	03/06/2021	**EGFR**	**EGFRp.(Val843Ile)**	WT	0.00	10.31	WT	0.00	12.05	EGFRp.(Val843Ile)	45.40	25.80
2030	DI	03/06/2021	**Other alterations**	**TP53p.(Cys176Phe)**	WT	0.00	59.25	NR	NR	NR	TP53p.(Cys176Phe)	3.20	107.80
2030	DI	03/06/2021	**KRAS**	**KRASp.(Gly12Val)**	KRASp.(Gly12Val)	6.10	59.25	NR	NR	NR	KRASp.(Gly12Val)	5.20	107.80
2032	DI	03/06/2021	**EGFR**	**EGFRp.(Leu747_Glu749del)**	EG-FRp.(Glu746_Ala750del)	1.10	77.50	NR	NR	NR	EG-FRp.(Leu747_Glu749del)	1.20	76.50
2036	P	25/07/2022	**Wild-type (WT)**	**Wild-type (WT)**	WT	0.00	36.88	WT	0.00	67.75	WT	0.00	150.00
2045	P	03/06/2021	**EGFR**	**EGFRp.(Thr790Met)**	WT	0.00	5.89	EGFRp.(Thr790Met)	0.05	9.35	WT	0.00	15.10
2045	P	03/06/2021	**EGFR**	**EGFRp.(Glu746_Ala750del)**	WT	0.00	5.89	EGFRp.(Glu746_Ala750del)	0.05	9.35	WT	0.00	15.10
2053	P	03/06/2021	**Other alterations**	**TP53p.(Arg110Leu)**	WT	0.00	17.50	WT	0.00	42.70	TP53p.(Arg110Leu)	6.10	44.20
2053	P	03/06/2021	**EGFR**	**EGFRp.(Gly719Ala)**	EGFRp.(Gly719Ala)	6.56	17.50	EGFRp.(Gly719X)	4.05	42.70	EGFRp.(Gly719Ala)	6.70	44.20
2061	P	03/06/2021	**Other alterations**	**CDKN2Ap.(Ser56Argfs*56)**	WT	0.00	79.38	NR	NR	NR	CDKN2Ap.(Ser56Ar-gfs*56)	9.70	150.00
2061	P	03/06/2021	**Other alterations**	**TP53p.(Arg283Cys)**	WT	0.00	79.38	NR	NR	NR	TP53p.(Arg283Cys)	56.50	150.00
2061	P	03/06/2021	**KRAS**	**KRASp.(Gly12Cys)**	KRASp.(Gly12Cys)	12.30	79.38	NR	NR	NR	KRASp.(Gly12Cys)	13.20	150.00
2066	DI	28/07/2022	**Wild-type (WT)**	**Wild-type (WT)**	WT	0.00	18.84	WT	0.00	7.42	WT	0.00	10.00
2069	P	02/08/2021	**EGFR**	**EGFRp.(Thr790Met)**	EGFRp.(Thr790Met)	0.60	16.00	EGFRp.(Thr790Met)	0.49	14.70	EGFRp.(Thr790Met)	2.00	18.00
2069	P	02/08/2021	**EGFR**	**EGFRp.(Leu861Gln)**	EGFRp.(Leu861Gln)	9.67	16.00	EGFRp.(Leu861Gln)	6.75	14.70	EGFRp.(Leu861Gln)	18.70	18.00
2103	DI	25/07/2022	**Other alterations**	**CTNNB1p.(Ala39_Ala43del)**	WT	0.00	36.88	WT	0.00	45.23	CTNNB1p.(Ala39_Ala43d el)	4.30	85.00
2103	DI	25/07/2022	**EGFR**	**EGFRp.(Glu746_Ala750del)**	EG-FRp.(Glu746_Ala750del)	8.48	36.88	EGFRp.(Glu746_Ala750del)	7.63	45.23	EG-FRp.(Glu746_Ala750del)	7.10	85.00
2105	P	03/06/2021	**EGFR**	**EGFRp.(Thr790Met)**	EGFRp.(Thr790Met)	0.17	35.81	EGFRp.(Thr790Met)	0.16	33.09	WT	0.00	77.00
2105	P	03/06/2021	**EGFR**	**EGFRp.(Leu718Gln)**	EGFRp.(Leu718Gln)	0.18	35.81	WT	0.00	33.09	WT	0.00	77.00
2105	P	03/06/2021	**EGFR**	**EGFRp.(Leu858Arg)**	EGFRp.(Leu858Arg)	0.26	35.81	EGFRp.(Leu858Arg)	0.22	33.09	EGFRp.(Leu858Arg)	0.30	77.00
2151	DI	02/08/2021	**PIK3CA**	**PIK3CAp.(Arg524Lys)**	WT	0.00	11.56	WT	0.00	22.28	PIK3CAp.(Arg524Lys)	1.40	35.00
2155	DI	25/07/2022	**Other alterations**	**TP53p.(Arg213*)**	WT	0.00	36.88	WT	0.00	48.53	TP53p.(Arg213*)	3.80	107.50
2155	DI	25/07/2022	**Other alterations**	**TP53p.(Arg333Valfs*12)**	WT	0.00	36.88	WT	0.00	48.53	TP53p.(Arg333Valfs*12)	9.20	107.50
2155	DI	25/07/2022	**EGFR**	**EGFRp.(Leu858Arg)**	EGFRp.(Leu858Arg)	65.47	36.88	EGFRp.(Leu858Arg)	45.22	48.53	EGFRp.(Leu858Arg)	68.70	107.50
2160	P	03/08/2022	**Wild-type (WT)**	**Wild-type (WT)**	WT	0.00	16.20	NR	NR	NR	WT	0.00	46.20
2165	P	28/07/2022	**Wild-type (WT)**	**Wild-type (WT)**	WT	0.00	8.46	WT	0.00	4.51	WT	0.00	8.70
2181	DI	18/07/2022	**EGFR**	**EGFRp.(Glu746_Ala750del)**	EG-FRp.(Glu746_Ala750del)	0.13	36.81	EGFRp.(Glu746_Ala750del)	0.10	88.20	WT	0.00	150.00
2224	DI	28/07/2022	**Wild-type (WT)**	**Wild-type (WT)**	WT	0.00	20.64	WT	0.00	21.36	WT	0.00	35.00
2227	DI	28/07/2022	**Wild-type (WT)**	**Wild-type (WT)**	WT	0.00	19.50	WT	0.00	17.28	WT	0.00	22.00
2229	DI	25/07/2022	**Other alterations**	**TP53p.(Cys135Tyr)**	WT	0.00	30.25	WT	0.00	31.60	TP53p.(Cys135Tyr)	1.40	54.00

2229	DI	25/07/2022	**KRAS**	**KRASp.(Gln61Arg)**	KRASp.(Gln61Arg)	0.25	30.25	WT	0.00	31.60	WT	0.00	54.00
2229	DI	25/07/2022	**EGFR**	**EGFRp.(Leu858Arg)**	EGFRp.(Leu858Arg)	3.69	30.25	EGFRp.(Leu858Arg)	2.25	31.60	EGFRp.(Leu858Arg)	5.20	54.00
2230	DI	18/07/2022	**EGFR**	**EGFRp.(Glu746_Ala750del)**	EG-FRp.(Glu746_Ala750del)	0.07	36.81	EGFRp.(Glu746_Ala750del)	0.13	44.15	WT	0.00	72.00
2238	DI	25/07/2022	**Wild-type (WT)**	**Wild-type (WT)**	WT	0.00	36.88	WT	0.00	54.01	WT	0.00	74.80
2249	P	25/07/2022	**Wild-type (WT)**	**Wild-type (WT)**	WT	0.00	33.90	NR	NR	NR	WT	0.00	28.30
2268	ND	03/06/2021	**EGFR**	**EGFRp.(Ser768Ile)**	EGFRp.(Ser768Ile)	2.37	25.98	WT	0.00	46.31	EGFRp.(Ser768Ile)	3.20	65.80
2268	ND	03/06/2021	**EGFR**	**EGFRp.(Gly719Ser)**	EGFRp.(Gly719Ser)	2.55	25.98	EGFRp.(Gly719X)	1.24	46.31	EGFRp.(Gly719Ser)	3.40	65.80
2275	P	18/07/2022	**Other alterations**	**TP53p.(Val274Ala)**	WT	0.00	18.29	NR	NR	NR	TP53p.(Val274Ala)	2.80	13.75
2275	P	18/07/2022	**EGFR**	**EGFRp.(Leu858Arg)**	EGFRp.(Leu858Arg)	2.04	18.29	NR	NR	NR	EGFRp.(Leu858Arg)	3.40	13.75
2291	DI	25/07/2022	**Wild-type (WT)**	**Wild-type (WT)**	WT	0.00	34.29	WT	0.00	24.44	WT	0.00	11.10
2312	ND	25/07/2022	**Wild-type (WT)**	**Wild-type (WT)**	WT	0.00	30.30	NR	NR	NR	WT	0.00	25.20
2324	DI	18/07/2022	**EGFR**	**EGFRp.(Glu746_Ala750del)**	EG-FRp.(Glu746_Ala750del)	38.61	36.81	EGFRp.(Glu746_Ala750del)	36.44	328.54	NR	NR	NR
2325	P	01/08/2022	**KRAS**	**KRASp.(Met72Leu)**	KRASp.(Met72Leu)	0.55	15.45	WT	0.00	12.37	KRASp.(Met72Leu)	1.10	19.00
2334	P	03/06/2021	**EGFR**	**EGFRp.(Cys797Ser)**	EGFRp.(Cys797Ser)	0.55	32.13	EGFRp.(Cys797Ser)	0.26	14.81	NR	NR	NR
2334	P	03/06/2021	**EGFR**	**EGFRp.(Glu746_Ala750del)**	EGFRp.L747_T751del	0.61	32.13	EGFRp.(Glu746_Ala750del)	0.66	14.81	NR	NR	NR
2334	P	03/06/2021	**EGFR**	**EGFRp.(Thr790Met)**	EGFRp.(Thr790Met)	1.02	32.13	EGFRp.(Thr790Met)	0.52	14.81	NR	NR	NR
2336	DI	25/07/2022	**PIK3CA**	**PIK3CAp.(Glu545Lys)**	PIK3CAp.(Glu545Lys)	27.58	36.88	WT	0.00	37.69	PIK3CAp.(Glu545Lys)	29.30	100.00
2336	DI	25/07/2022	**EGFR**	**EGFRp.(Glu746_Ala750del)**	EG-FRp.(Glu746_Ala750del)	64.04	36.88	EGFRp.(Glu746_Ala750del)	60.41	37.69	EG-FRp.(Glu746_Ala750del)	62.20	100.00
2338	DI	03/06/2021	**Other alterations**	**TP53p.(Val173Leu)**	WT	0.00	82.50	WT	0.00	106.58	TP53p.(Val173Leu)	24.60	100.00
2338	DI	03/06/2021	**EGFR**	**EGFRp.(Gly779Cys)**	WT	0.00	82.50	WT	0.00	106.58	EGFRp.(Gly779Cys)	31.70	100.00
2338	DI	03/06/2021	**EGFR**	**EGFRp.(Leu861Gln)**	EGFRp.(Leu861Gln)	29.04	82.50	EGFRp.(Leu861Gln)	20.31	106.58	EGFRp.(Leu861Gln)	31.50	100.00
2377	P	18/07/2022	**EGFR**	**EGFRp.(Glu746_Ala750del)**	EG-FRp.(Glu746_Ala750del)	0.02	36.81	EGFRp.(Glu746_Ala750del)	0.03	36.43	WT	0.00	72.30
2379	P	03/08/2022	**EGFR**	**EGFRp.(Thr790Met)**	WT	0.00	6.75	EGFRp.(Thr790Met)	0.01	14.02	WT	0.00	25.80
2379	P	03/08/2022	**EGFR**	**EGFRp.(Glu746_Ala750del)**	WT	0.00	6.75	EGFRp.(Glu746_Ala750del)	0.05	14.02	WT	0.00	25.80
2387	P	25/07/2022	**BRAF**	**BRAFp.(Asp594Asn)**	WT	0.00	36.88	NR	NR	NR	BRAFp.(Asp594Asn)	1.50	37.50
2428	P	17/08/2022	**EGFR**	**EGFRp.(Leu718Gln)**	EGFRp.(Leu718Gln)	0.52	37.50	WT	0.00	91.04	WT	0.00	100.00
2428	P	17/08/2022	**EGFR**	**EGFRp.(Thr790Met)**	EGFRp.(Thr790Met)	0.83	37.50	EGFRp.(Thr790Met)	0.49	91.04	WT	0.00	100.00
2428	P	17/08/2022	**EGFR**	**EGFRp.(Leu858Arg)**	EGFRp.(Leu858Arg)	1.41	37.50	EGFRp.(Leu858Arg)	0.59	91.04	EGFRp.(Leu858Arg)	1.60	100.00
2441	DI	24/08/2021	**KRAS**	**KRASp.(Gly12Cys)**	WT	0.00	3.44	WT	0.00	2.66	KRASp.(Gly12Cys)	0.50	8.49
2452	DI	01/08/2022	**Wild-type (WT)**	**Wild-type (WT)**	WT	0.00	8.37	WT	0.00	18.63	WT	0.00	32.00
2453	DI	02/08/2021	**BRAF**	**BRAFp.(Val600Glu)**	BRAFp.(Val600Glu)	0.59	6.24	WT	0.00	10.39	BRAFp.(Val600Glu)	0.60	22.10
2454	DI	24/08/2021	**KRAS**	**KRASp.(Gln61Leu)**	KRASp.(Gln61Leu)	58.96	36.25	NR	NR	NR	KRASp.(Gln61Leu)	67.50	150.00
2455	DI	24/08/2021	**Other alterations**	**METp.(?)**	WT	0.00	16.87	NR	0.00	18.92	METp.(?)	1.50	34.80
2455	DI	24/08/2021	**Other alterations**	**METp.(Leu1213Phe)**	WT	0.00	16.87	WT	0.00	18.92	METp.(Leu1213Phe)	5.90	34.80
2455	DI	24/08/2021	**Other alterations**	**TP53p.(Glu294Glyfs*12)**	WT	0.00	16.87	WT	0.00	18.92	TP53p.(Glu294Glyfs*12)	11.30	34.80
2455	DI	24/08/2021	**EGFR**	**EGFRp.(Ile744.)**	EGFRp.(Ile744.)	1.40	16.87	WT	0.00	18.92	WT	0.00	34.80
2455	DI	24/08/2021	**PIK3CA**	**PIK3CAp.(Glu542Lys)**	PIK3CAp.(Glu545Lys)	1.90	16.87	WT	0.00	18.92	PIK3CAp.(Glu542Lys)	2.80	34.80
2460	DI	24/08/2021	**BRAF**	**BRAFp.(Val600Glu)**	BRAFp.(Val600Glu)	0.50	15.62	WT	0.00	30.51	BRAFp.(Val600Glu)	0.70	30.30
2460	DI	24/08/2021	**PIK3CA**	**PIK3CAp.(Glu545Lys)**	PIK3CAp.(Glu545Lys)	0.70	15.62	WT	0.00	30.51	PIK3CAp.(Glu545Lys)	2.10	30.30
2462	DI	01/08/2022	**Wild-type (WT)**	**Wild-type (WT)**	WT	0.00	7.05	WT	0.00	10.29	WT	0.00	14.70
2463	DI	24/08/2021	**KRAS**	**KRASp.(Gly12Val)**	KRASp.(Gly12Val)	1.20	26.87	WT	0.00	25.65	KRASp.(Gly12Val)	1.40	27.00
2464	P	24/08/2021	**EGFR**	**EGFRp.(Thr790Met)**	EGFRp.(Thr790Met)	1.20	85.00	EGFRp.(Thr790Met)	0.77	411.77	WT	0.00	150.00
2464	P	24/08/2021	**EGFR**	**EGFRp.(Leu858Arg)**	EGFRp.(Leu858Arg)	4.80	85.00	EGFRp.(Leu858Arg)	2.80	411.77	EGFRp.(Leu858Arg)	5.00	150.00
2466	DI	01/08/2022	**Wild-type (WT)**	**Wild-type (WT)**	WT	0.00	7.68	WT	0.00	16.90	WT	0.00	18.70
2471	P	25/07/2022	**Wild-type (WT)**	**Wild-type (WT)**	WT	0.00	36.88	NR	NR	NR	WT	0.00	33.50
2472	P	24/08/2021	**Other alterations**	**TP53p.(?)**	WT	0.00	85.00	NR	NR	NR	TP53p.(?)	8.10	93.00
2472	P	24/08/2021	**BRAF**	**BRAFp.(Val600Glu)**	BRAFp.(Val600Glu)	7.70	85.00	NR	NR	NR	BRAFp.(Val600Glu)	5.10	93.00
2473	DI	25/07/2022	**Wild-type (WT)**	**Wild-type (WT)**	WT	0.00	36.88	WT	0.00	124.07	WT	0.00	100.00
2476	P	28/07/2022	**EGFR**	**EGFRp.(Leu718Gln)**	EGFRp.(Leu718Gln)	0.18	36.81	WT	0.00	31.88	WT	0.00	100.00
2476	P	28/07/2022	**EGFR**	**EGFRp.(Thr790Met)**	EGFRp.(Thr790Met)	0.43	36.81	EGFRp.(Thr790Met)	0.35	31.88	WT	0.00	100.00
2476	P	28/07/2022	**EGFR**	**EGFRp.(Leu858Arg)**	EGFRp.(Leu858Arg)	0.61	36.81	EGFRp.(Leu858Arg)	0.30	31.88	WT	0.00	100.00
2477	DI	25/07/2022	**Wild-type (WT)**	**Wild-type (WT)**	WT	0.00	36.88	WT	0.00	30.30	WT	0.00	92.20
2478	DI	25/07/2022	**Wild-type (WT)**	**Wild-type (WT)**	WT	0.00	36.88	WT	0.00	220.44	WT	0.00	100.00
2481	DI	25/07/2022	**Wild-type (WT)**	**Wild-type (WT)**	WT	0.00	31.25	WT	0.00	29.16	WT	0.00	50.00
2482	DI	24/08/2021	**KRAS**	**KRASp.(Gly12Asp)**	KRASp.(Gly12Asp)	1.20	16.75	NR	NR	NR	KRASp.(Gly12Asp)	1.60	16.75
2482	DI	24/08/2021	**BRAF**	**BRAFp.(Asn581Ile)**	BRAFp.(Asn581Ile)	1.40	16.75	NR	NR	NR	BRAFp.(Asn581Ile)	1.40	16.75

2483	DI	18/07/2022	**Wild-type (WT)**	**Wild-type (WT)**	WT	0.00	36.81	WT	0.00	37.32	WT	0.00	50.00
2485	P	02/08/2021	**Other alterations**	**TP53p.(Arg248Trp)**	WT	0.00	10.05	NR	NR	NR	TP53p.(Arg248Trp)	1.60	50.00
2485	P	02/08/2021	**KRAS**	**KRASp.(Gly12Cys)**	KRASp.(Gly12Cys)	4.73	10.05	NR	NR	NR	KRASp.(Gly12Cys)	5.80	50.00
2486	P	24/08/2021	**EGFR**	**EGFRp.(Cys797Ser)**	EGFRp.(Cys797Ser)	0.20	36.25	EGFRp.(Cys797Ser)	0.16	67.17	WT	0.00	84.40
2486	P	24/08/2021	**EGFR**	**EGFRp.(Thr790Met)**	EGFRp.(Thr790Met)	0.30	36.25	EGFRp.(Thr790Met)	0.24	67.17	WT	0.00	84.40
2486	P	24/08/2021	**EGFR**	**EGFRp.(Glu746_Ala750del)**	EG-<br>FRp.(Glu746_Ala750del)	1.10	36.25	EGFRp.(Glu746_Ala750del)	1.29	67.17	EG-<br>FRp.(Glu746_Ala750del)	1.40	84.40
2490	DI	03/08/2022	**Wild-type (WT)**	**Wild-type (WT)**	WT	0.00	18.30	NR	NR	NR	WT	0.00	15.30
2497	P	17/08/2022	**EGFR**	**EGFRp.(Leu858Arg)**	EGFRp.(Leu858Arg)	0.28	23.66	EGFRp.(Leu858Arg)	0.15	23.05	WT	0.00	28.30
2497	P	17/08/2022	**KRAS**	**KRASp.(Met72Leu)**	KRASp.(Met72Leu)	0.97	23.66	WT	0.00	23.05	KRASp.(Met72Leu)	1.50	28.30
2547	P	17/08/2022	**EGFR**	**EGFRp.(Leu858Arg)**	EGFRp.(Leu858Arg)	0.30	37.50	EGFRp.(Leu858Arg)	0.17	39.47	WT	0.00	37.00
2547	P	17/08/2022	**KRAS**	**KRASp.(Met72Leu)**	KRASp.(Met72Leu)	0.68	37.50	WT	0.00	39.47	WT	0.00	37.00
2549	P	01/08/2022	**Wild-type (WT)**	**Wild-type (WT)**	WT	0.00	7.62	WT	0.00	5.42	WT	0.00	9.80
2579	ND	12/08/2022	**Wild-type (WT)**	**Wild-type (WT)**	WT	0.00	25.00	NR	NR	NR	WT	0.00	22.50
2599	P	12/08/2022	**Wild-type (WT)**	**Wild-type (WT)**	WT	0.00	36.81	NR	NR	NR	WT	0.00	32.50
2652	P	12/08/2022	**Wild-type (WT)**	**Wild-type (WT)**	WT	0.00	36.81	NR	NR	NR	WT	0.00	34.00
2700	P	18/07/2022	**EGFR**	**EGFRp.(Leu861Gln)**	EGFRp.(Leu861Gln)	0.35	36.81	EGFRp.(Leu861Gln)	0.22	39.92	WT	0.00	52.30
2708	DI	01/08/2022	**Wild-type (WT)**	**Wild-type (WT)**	WT	0.00	4.44	WT	0.00	16.80	WT	0.00	19.40
2710	DI	28/07/2022	**Wild-type (WT)**	**Wild-type (WT)**	WT	0.00	19.81	WT	0.00	45.81	WT	0.00	41.10
2711	P	28/07/2022	**Wild-type (WT)**	**Wild-type (WT)**	WT	0.00	12.09	WT	0.00	6.40	WT	0.00	10.32
2724	P	18/07/2022	**EGFR**	**EGFRp.(Ser768Ile)**	EGFRp.(Ser768Ile)	0.39	31.25	WT	0.00	43.13	WT	0.00	66.00
2724	P	18/07/2022	**EGFR**	**EGFRp.(Gly719Ser)**	EGFRp.(Gly719Ser)	0.45	31.25	EGFRp.(Gly719Ser)	0.12	43.13	EGFRp.(Gly719Ser)	0.50	66.00
2791	P	12/08/2022	**Wild-type (WT)**	**Wild-type (WT)**	WT	0.00	36.81	NR	NR	NR	WT	0.00	59.30
2824	P	12/08/2022	**Wild-type (WT)**	**Wild-type (WT)**	WT	0.00	31.38	NR	NR	NR	WT	0.00	28.60
2849	P	03/08/2022	**Wild-type (WT)**	**Wild-type (WT)**	WT	0.00	15.30	NR	NR	NR	WT	0.00	13.25
2850	P	03/08/2022	**Wild-type (WT)**	**Wild-type (WT)**	WT	0.00	15.00	NR	NR	NR	WT	0.00	12.99
2856	P	03/08/2022	**Wild-type (WT)**	**Wild-type (WT)**	WT	0.00	16.80	NR	NR	NR	WT	0.00	14.60
2857	P	12/08/2022	**Wild-type (WT)**	**Wild-type (WT)**	WT	0.00	36.81	NR	NR	NR	WT	0.00	100.00
2859	DI	12/08/2022	**Wild-type (WT)**	**Wild-type (WT)**	WT	0.00	36.81	NR	NR	NR	WT	0.00	31.20
2860	P	03/08/2022	**Wild-type (WT)**	**Wild-type (WT)**	WT	0.00	9.00	NR	NR	NR	WT	0.00	27.00
2861	DI	12/08/2022	**Wild-type (WT)**	**Wild-type (WT)**	WT	0.00	36.81	NR	NR	NR	WT	0.00	48.45
2862	P	12/08/2022	**Wild-type (WT)**	**Wild-type (WT)**	WT	0.00	36.81	NR	NR	NR	WT	0.00	100.00
2864	DI	12/08/2022	**Wild-type (WT)**	**Wild-type (WT)**	WT	0.00	36.81	NR	NR	NR	WT	0.00	41.10
2865	DI	12/08/2022	**Wild-type (WT)**	**Wild-type (WT)**	WT	0.00	34.63	NR	NR	NR	WT	0.00	36.60
2866	P	03/08/2022	**Wild-type (WT)**	**Wild-type (WT)**	WT	0.00	18.00	NR	NR	NR	WT	0.00	15.50
2867	P	12/08/2022	**Wild-type (WT)**	**Wild-type (WT)**	WT	0.00	34.63	NR	NR	NR	WT	0.00	28.10
2870	DI	12/08/2022	**Wild-type (WT)**	**Wild-type (WT)**	WT	0.00	33.75	NR	NR	NR	WT	0.00	28.90
2871	DI	12/08/2022	**Wild-type (WT)**	**Wild-type (WT)**	WT	0.00	36.81	NR	NR	NR	WT	0.00	58.20
2873	P	03/08/2022	**Wild-type (WT)**	**Wild-type (WT)**	WT	0.00	18.30	NR	NR	NR	WT	0.00	15.75
2874	P	03/08/2022	**Wild-type (WT)**	**Wild-type (WT)**	WT	0.00	18.30	NR	NR	NR	WT	0.00	15.75
2875	P	12/08/2022	**Wild-type (WT)**	**Wild-type (WT)**	WT	0.00	36.81	NR	NR	NR	WT	0.00	38.50
2876	DI	03/08/2022	**KRAS**	**KRASp.(Lys117Asn)**	KRASp.(Lys117Asn)	0.35	17.40	NR	NR	NR	WT	0.00	15.10
2877	P	17/08/2022	**Wild-type (WT)**	**Wild-type (WT)**	WT	0.00	24.39	NR	NR	NR	WT	0.00	19.80
2878	DI	17/08/2022	**Wild-type (WT)**	**Wild-type (WT)**	WT	0.00	39.06	NR	NR	NR	WT	0.00	32.80
2892	DI	03/08/2022	**Wild-type (WT)**	**Wild-type (WT)**	WT	0.00	14.40	NR	NR	NR	WT	0.00	12.50
2893	P	17/08/2022	**Other alterations**	**NOTCH1p.(Pro2415del)**	WT	0.00	33.99	NR	NR	NR	NOTCH1p.(Pro2415del)	3.10	26.80
2899	P	28/07/2022	**Wild-type (WT)**	**Wild-type (WT)**	WT	0.00	8.25	WT	0.00	6.81	WT	0.00	12.20
2904	P	01/08/2022	**Wild-type (WT)**	**Wild-type (WT)**	WT	0.00	13.53	WT	0.00	15.63	WT	0.00	26.10
2905	P	01/08/2022	**Other alterations**	**TP53p.(Arg158Pro)**	WT	0.00	36.81	WT	0.00	153.04	TP53p.(Arg158Pro)	37.20	100.00
2906	DI	01/08/2022	**Wild-type (WT)**	**Wild-type (WT)**	WT	0.00	4.38	WT	0.00	25.42	WT	0.00	63.60
2908	DI	01/08/2022	**Wild-type (WT)**	**Wild-type (WT)**	WT	0.00	12.33	WT	0.00	17.72	WT	0.00	30.40
2909	P	17/08/2022	**Other alterations**	**TP53p.(Val157Phe)**	WT	0.00	37.50	NR	NR	NR	TP53p.(Val157Phe)	1.80	100.00
2909	P	17/08/2022	**EGFR**	**EGFRp.(Leu833Phe)**	WT	0.00	37.50	NR	NR	NR	EGFRp.(Leu833Phe)	85.60	100.00
2909	P	17/08/2022	**EGFR**	**EGFRp.(Cys797Ser)**	EGFRp.(Cys797Ser)	0.20	37.50	NR	NR	NR	WT	0.00	100.00
2909	P	17/08/2022	**EGFR**	**EGFRp.(Thr790Met)**	EGFRp.(Thr790Met)	1.00	37.50	NR	NR	NR	EGFRp.(Thr790Met)	1.10	100.00
2909	P	17/08/2022	**EGFR**	**EGFRp.(Leu861Gln)**	EGFRp.(Leu861Gln)	82.25	37.50	NR	NR	NR	EGFRp.(Leu861Gln)	85.00	100.00
2910	DI	17/08/2022	**Wild-type (WT)**	**Wild-type (WT)**	WT	0.00	37.50	NR	NR	NR	WT	0.00	31.70
2911	DI	17/08/2022	**Wild-type (WT)**	**Wild-type (WT)**	WT	0.00	37.50	NR	NR	NR	WT	0.00	100.00
2912	DI	17/08/2022	**KRAS**	**KRASp.(Gly12Asp)**	KRASp.(Gly12Asp)	0.29	37.50	NR	NR	NR	WT	0.00	100.00
2914	DI	17/08/2022	**Wild-type (WT)**	**Wild-type (WT)**	WT	0.00	37.50	NR	NR	NR	WT	0.00	61.00

2915	DI	17/08/2022	**Other alterations**	**TP53p.(His179Tyr)**	WT	0.00	37.50	NR	NR	NR	TP53p.(His179Tyr)	26.20	36.20
2916	P	17/08/2022	**Wild-type (WT)**	**Wild-type (WT)**	WT	0.00	33.30	NR	NR	NR	WT	0.00	28.90
2917	P	18/07/2022	**BRAF**	**BRAFp.(Val600Glu)**	BRAFp.(Val600Glu)	1.00	36.81	NR	NR	NR	BRAFp.(Val600Glu)	1.50	100.00
2917	P	18/07/2022	**PIK3CA**	**PIK3CAp.(Glu545Lys)**	PIK3CAp.(Glu545Lys)	1.22	36.81	NR	NR	NR	PIK3CAp.(Glu545Lys)	1.80	100.00
2918	DI	18/07/2022	**Wild-type (WT)**	**Wild-type (WT)**	WT	0.00	31.25	NR	NR	NR	WT	0.00	27.30
2919	P	18/07/2022	**Other alterations**	**TP53p.(Ile255Phe)**	WT	0.00	36.81	NR	NR	NR	TP53p.(Ile255Phe)	1.60	59.70
2919	P	18/07/2022	**Other alterations**	**GNASp.(Arg201His)**	WT	0.00	36.81	NR	NR	NR	GNASp.(Arg201His)	7.90	59.70
2919	P	18/07/2022	**EGFR**	**EGFRp.(Leu861Gln)**	EGFRp.(Leu861Gln)	4.00	36.81	NR	NR	NR	EGFRp.(Leu861Gln)	4.90	59.70
2920	DI	30/06/2022	**Wild-type (WT)**	**Wild-type (WT)**	WT	0.00	36.25	NR	NR	NR	WT	0.00	100.00
2921	P	30/06/2022	**Other alterations**	**TP53p.(Gln331*)**	WT	0.00	36.25	NR	NR	NR	TP53p.(Gln331*)	8.10	73.70
2921	P	30/06/2022	**PIK3CA**	**PIK3CAp.(Glu545Lys)**	PIK3CAp.(Glu545Lys)	2.00	36.25	NR	NR	NR	PIK3CAp.(Glu545Lys)	2.50	73.70
2922	P	30/06/2022	**Wild-type (WT)**	**Wild-type (WT)**	WT	0.00	25.00	NR	NR	NR	WT	0.00	30.40
2923	P	30/06/2022	**Wild-type (WT)**	**Wild-type (WT)**	WT	0.00	36.25	NR	NR	NR	WT	0.00	47.80
2924	P	30/06/2022	**Wild-type (WT)**	**Wild-type (WT)**	WT	0.00	25.00	NR	NR	NR	WT	0.00	26.80
2926	DI	30/06/2022	**Wild-type (WT)**	**Wild-type (WT)**	WT	0.00	36.25	NR	NR	NR	WT	0.00	72.80
2927	DI	30/06/2022	**Wild-type (WT)**	**Wild-type (WT)**	WT	0.00	36.25	NR	NR	NR	WT	0.00	77.80
2928	DI	30/06/2022	**Wild-type (WT)**	**Wild-type (WT)**	WT	0.00	25.00	NR	NR	NR	WT	0.00	25.30
2929	DI	30/06/2022	**Other alterations**	**TP53p.(Asn131del)**	WT	0.00	36.25	NR	NR	NR	TP53p.(Asn131del)	38.70	100.00
2930	P	30/06/2022	**BRAF**	**BRAFp.(Val600Glu)**	BRAFp.(Val600Glu)	33.00	36.25	NR	NR	NR	BRAFp.(Val600Glu)	33.80	100.00
2931	P	30/06/2022	**Other alterations**	**TP53p.(Glu224*)**	WT	0.00	36.25	NR	NR	NR	TP53p.(Glu224*)	5.40	57.70
2932	DI	30/06/2022	**Other alterations**	**TP53p.(Ile255del)**	WT	0.00	36.25	NR	NR	NR	TP53p.(Ile255del)	1.60	75.00
2932	DI	30/06/2022	**KRAS**	**KRASp.(Gly12Cys)**	KRASp.(Gly12Cys)	2.00	36.25	NR	NR	NR	KRASp.(Gly12Cys)	2.20	75.00
2933	DI	30/06/2022	**Wild-type (WT)**	**Wild-type (WT)**	WT	0.00	36.25	NR	NR	NR	WT	0.00	100.00
2934	P	30/06/2022	**Other alterations**	**TP53p.(Val172Phe)**	WT	0.00	20.00	NR	NR	NR	TP53p.(Val172Phe)	0.40	20.50
2934	P	30/06/2022	**EGFR**	**EGFRp.(Gly719Cys)**	EGFRp.(Gly719Cys)	0.00	20.00	NR	NR	NR	EGFRp.(Gly719Cys)	0.50	20.50
2935	DI	30/06/2022	**Other alterations**	**TP53p.(Arg248Trp)**	WT	0.00	25.00	NR	NR	NR	TP53p.(Arg248Trp)	2.50	30.40
2935	DI	30/06/2022	**KRAS**	**KRASp.(Gly12Cys)**	KRASp.(Gly12Cys)	4.00	25.00	NR	NR	NR	KRASp.(Gly12Cys)	4.20	30.40
2937	DI	23/06/2022	**Other alterations**	**H3F3Ap.(Ser29Thr)**	WT	0.00	12.50	NR	NR	NR	H3F3Ap.(Ser29Thr)	13.20	20.50
2938	DI	23/06/2022	**Wild-type (WT)**	**Wild-type (WT)**	WT	0.00	36.25	NR	NR	NR	WT	0.00	53.80
2939	DI	23/06/2022	**Wild-type (WT)**	**Wild-type (WT)**	WT	0.00	36.25	NR	NR	NR	WT	0.00	38.00
2940	P	23/06/2022	**Wild-type (WT)**	**Wild-type (WT)**	WT	0.00	25.00	NR	NR	NR	WT	0.00	42.00
2941	DI	23/06/2022	**Other alterations**	**TP53p.(Ser215Gly)**	WT	0.00	16.75	NR	NR	NR	TP53p.(Ser215Gly)	3.00	28.30
2941	DI	23/06/2022	**Other alterations**	**TP53p.(Cys275Tyr)**	WT	0.00	16.75	NR	NR	NR	TP53p.(Cys275Tyr)	9.10	28.30
2941	DI	23/06/2022	**EGFR**	**EGFRp.(Leu858Arg)**	EGFRp.(Leu858Arg)	12.00	16.75	NR	NR	NR	EGFRp.(Leu858Arg)	12.30	28.30
2942	P	23/06/2022	**BRAF**	**BRAFp.(Val600Glu)**	BRAFp.(Val600Glu)	2.00	36.25	NR	NR	NR	BRAFp.(Val600Glu)	2.30	100.00
2943	DI	23/06/2022	**KRAS**	**KRASp.(Gly13Val)**	KRASp.(Gly13Val)	1.00	20.00	NR	NR	NR	KRASp.(Gly13Val)	0.60	62.10
1599	DI	20/07/2022	**EGFR**	**EGFRp.(Glu746_Ala750del)**	EG-FRp.(Glu746_Ala750del)	20.02	36.81	EGFRp.(Glu746_Ala750del)	14.14	71.17	EG-FRp.(Glu746_Ala750del)	10.60	50.00
